# Highly Sensitive AgNP/MWCNT/Nafion Modified GCE-Based Sensor for the Determination of Heavy Metals in Organic and Non-organic Vegetables

**DOI:** 10.1038/s41598-018-35781-x

**Published:** 2018-11-28

**Authors:** Shirley Tiong Palisoc, Michelle Tiamzon Natividad, Nico De Jesus, Joshua Carlos

**Affiliations:** 1 Physics Department, De La Salle University, Manila, Philippines; 2 Condensed Matter Research Unit, CENSER, De La Salle University, Manila, Philippines

## Abstract

Silver nanoparticles/multi-walled carbon nanotubes/Nafion modified glassy carbon electrodes (AgNPs/MWCNTs/Nafion-GCE) were fabricated and were used as working electrode in anodic stripping voltammetry (ASV) for trace level determination of lead (Pb^2+^) and cadmium (Cd^2+^). The fabricated electrodes were characterized using scanning electron microscopy and cyclic voltammetry. The amounts of the electrode modifiers and the ASV parameters were optimized. It was found that the electrode modified with 1 mg AgNPs and 2 mg MWCNTs exhibited the best analytical response towards the determination of Pb^2+^ and Cd^2+^. The optimized ASV parameters were 60 s for the deposition time, 90 s for the accumulation time, and 100 mV/s for the scan rate. The electrode exhibited linearity from 0.493 ppb to 157.2 ppb for Pb^2+^ and 1.864 ppb to 155.1 ppb for Cd^2+^. The limit of detection was found to be 0.216 ppb for Pb^2+^ and 0.481 ppb for Cd^2+^. Real sampling analysis was carried out using organic vegetables from Sitio San Ysiro, Antipolo and Daraitan, Rizal and commercially available vegetables from Divisoria, all in Luzon, Philippines. Trace amounts of lead, cadmium, and copper were detected in the samples. Unwashed vegetables contained more heavy metal concentration compared to the washed vegetables. Atomic absorption spectroscopy was performed to validate the presence of the heavy metals in the vegetables.

## Introduction

Vegetables are one of the cornerstones of human nutrition^1^. They contain different vitamins and phytochemicals such as vitamins A, D and E, iron, antioxidants, phytoestrogens, and anti-inflammatory agents^2^. The Food and Nutrition Research Institute of the Philippines (FNRI) recommends that an average adult Filipino (19–59 years old) must at least have 1 cup of vegetables in every meal^3^. Studies, however, show that high concentrations of heavy metals such as zinc, copper, lead, and cadmium are present in vegetables^4–12^. Different factors of heavy metal contamination in vegetables may be attributed to pesticides, mine tailings and pollution of soil, water, as well as air^11^. Illegal mining in the Philippines is very extensive. An example of which are the illegal gold panning activities in San Ysiro, Antipolo^13^. These illegal mining activities may lead to the trace heavy metal contamination in vegetables planted in the said area.

Trace heavy metals are absorbed by plants through different pathways. Cadmium (Cd^2+^) enters the plant through the roots and then transported and stored to the stem through the low affinity cation transporter which is also responsible for the calcium transport in plants. Lead (Pb^2+^), on the other hand, binds with the carboxylic groups of the mucilage uronic acids on the root surfaces and subsequently enters the plant. High concentrations of Pb^2+^ are found to be concentrated on the phloem which suggests that it moves from the xylem to the leaves of the plants.

High concentrations of heavy metals are harmful to the body especially when ingested^2^. The World Health Organization (WHO) established that the limits for Cd^2+^ and Pb^2+^ are 200 ppb and 300 ppb, respectively^14^. These toxic metals may be absorbed by the vegetables through different processes and enter the food chain at high concentrations which may cause health risks for the consumers. When these metals enter the body, they may be stored in different organs of the body like the kidneys, blood, lungs, liver, and other vital organs^15^. These metals can cause harmful effects to the nervous, cardiovascular, and reproductive systems. Chronic exposure to these metals may even lead to death^5^. The alarming presence of heavy metals in vegetables warrants an urgent need to determine their heavy metal concentration. Anodic stripping voltammetry (ASV) is one of the different methods used in measuring heavy metal concentrations in matter. This technique uses three electrodes, namely, a working electrode, a reference electrode and a counter electrode. The working electrode acts as a sensor in the analysis of different analytes in different matrices^6^. The performance of this sensor is strongly influenced by the working electrode material. Chemical modification of the working electrode surface by nanomaterials (e.g. graphene, carbon nanotubes, silver nanoparticles (AgNPs), gold nanoparticles) is done to enhance its sensitivity. Silver nanoparticles are proven to be excellent for modifying electrodes due to their high conductivity and high stability^16^. Different studies suggest that modified electrodes with AgNPs showed better limits of detection^17,18^. Multi-walled carbon nanotubes (MWCNTs) exhibit remarkable electrochemical properties because they have more electrochemically active sites, making them very attractive for electrochemical determination of heavy metals at low potentials^19,20^.

In this study, silver nanoparticles/multi-walled carbon nanotubes/Nafion modified glassy carbon electrodes (AgNPs/MWCNTs/Nafion-GCE) were fabricated via the drop coating technique. The modified electrodes were used to detect traces of heavy metal ions in vegetables via anodic stripping voltammetry.

## Methodology

### Materials and Equipment

The following materials and equipment were used in the study: GCE, BOSCH SAE200 electronic balance, micro pipette, ROCKER ultrasonic bath sonicator, beakers. The chemicals and reagents for the fabrication of the AgNPs/MWCNTs/Nafion modified GCE required AgNPs, MWCNTs, Nafion, ethanol, aluminum slurry and deionized water. Cadmium chloride and lead chloride were used to produce stock solutions for the calibration curve of Cd^2+^ and Pb^2+^ and for the determination of the optimization parameters of the modified electrode. The cadmium chloride, lead chloride, AgNPs, and MWCNTs were procured from Sigma Aldrich.

### Preparation of the GCE and the Stock Solution

The fabrication of the modified electrode was accomplished by drop coating onto the polished tip of the GCE the modifier solution consisting of AgNPs, MWCNT and Nafion. The amounts of AgNPs and MWCNTs were varied to determine the best modified GCE. The amount of MWCNT was set at 1, 2, and 3 mg per 5 ml of Nafion. The amount of the AgNPs was varied at 1, 2, and 3 mg per concentration of MWCNT. The AgNPs and MWCNT were added to Nafion and the resulting solution was sonicated to homogenize the mixture. The modifier solution (5 μL) was then deposited onto the bare GCE surface using a micropipette. The modified electrodes were air-dried at room temperature (25 °C) for 2 hours.

Reagents for the determination of the optimization of ASV parameters include 10 ppm of CdCl_2_ and PbCl_2_. A concentration of 1.3 mg of PbCl_2_ and 1.6 mg of CdCl_2_ were added to the analyte mixture containing 0.1 M of NaCl. The calibration curve measurements of the individual metals required a stock solution containing 10 ppm of CdCl_2_ and PbCl_2_ were prepared.

### Real Sample Analysis

#### Vegetable and soil sampling

The study made use of organic vegetables grown in Sitio San Ysiro, Antipolo City and Daraitan, Rizal as well as non-organic vegetables procured commercially from Divisoria. Trace heavy metals in organic and non-organic vegetables were determined using the modified electrodes fabricated in this study. The origin of the trace heavy metal contamination was determined.

The sample vegetables were Bok Choy (*Brassica rapa subsp*. *chinensis*), cabbage (*Brassica oleracea var*. *capitata*), camote tops (*Ipomoea batatas*), and kabocha (*Cucurbita maxima*). All the samples were chemically digested. Heavy metal contamination was determined in both washed and unwashed vegetable samples to distinguish contamination from environmental pollutants; i.e., from air pollutants plus water as well as soil contamination or from water and soil contamination only. The washed vegetables were obtained by rinsing the samples with running water for 5 minutes.

#### Site Description

Sitio San Ysiro is situated in a valley within the lower Sierra Madre mountain range and is about a two-hour drive from Metro Manila. Daraitan, a barangay of Tanay, Rizal is situated southeast of Sitio San Ysiro. The coordinates of San Ysiro are: Latitude: 14.7°N, Longitude: 121.3°E, 764 m above sea level; whereas that of Daraitan are: Latitude: 14.6°N, Longitude: 121.4°E, 764 m above sea level.

Both sites for organic samples are far from main roads and urbanization with mainly rice and vegetable farming as the main source of livelihood. Some farmers practice slash and burn farming (kaingin). Irrigation is sourced from springs and nearby streams. Their water sources are far or upstream from residential areas or animal farms. However, one potential heavy metal source is the presence of small-scale mining (gold panning) in some parts of the river.

### Acid Digestion and Dry Ashing of Vegetable Samples

The vegetables were chopped to small pieces and weighed. A sample of about 20 mg was put in a 30 to 40 g ceramic crucible and placed inside the 48000 Thermolyne muffle furnace for 6 to 12 hours, depending on the vegetable sample, at 450 °C to achieve a white ash. The resulting white ash was added with 4 mL of HCl and was allowed to evaporate; after which, 100 mL of deionized water was added to the sample and was filtered. Approximately 0.6 g of NaCl was added to the resulting analyte.

### Acid Digestion of Soil Sample

Prior to weighing, non-soil particles were removed using a pair of laboratory tweezers. One g of soil was put in a beaker and 10 mL of nitric acid was added to the sample. The crucible containing the sample was heated until the solution becomes completely clear and solid traces of the soil sample were no longer visible. The solution was diluted in 100 mL deionized water and approximately 0.6 g of NaCl was added to the resulting analyte.

### Anodic Stripping Voltammetry

In order determine the heavy metal content of the vegetable samples, anodic stripping voltammetry was utilized. The electronic work station consists of a voltammetry cell and an Autolab potentiostat interfaced to a computer. The voltammetric cell consists of a working electrode, a reference electrode and a counter electrode. The fabricated electrode was used as the working electrode to interact with the analyte, Ag/AgCl was used as the reference electrode and a platinum coil was used as the counter electrode.

## Results and Discussion

### Determination of the Best Modified Electrode

**Figure 1:**
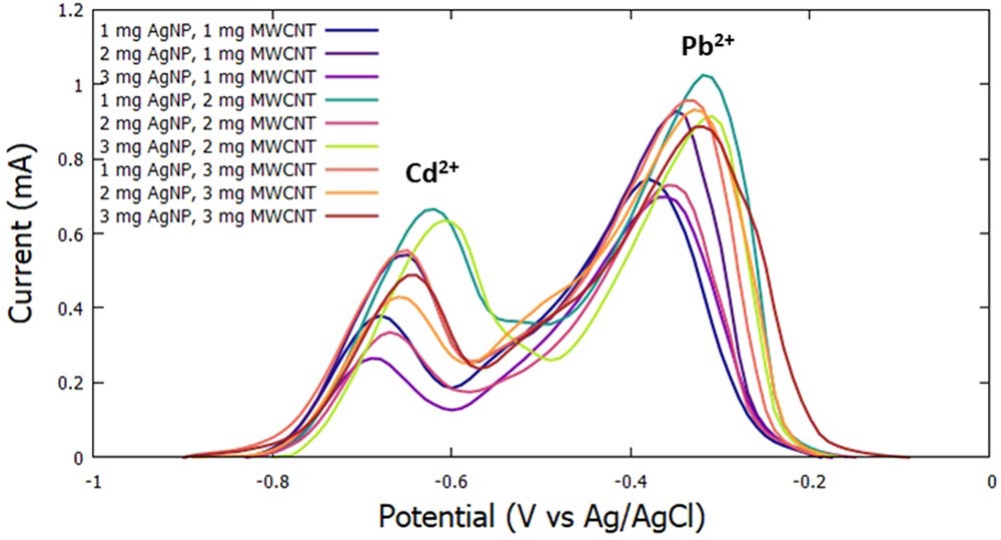
Voltammograms for varying MWCNT and AgNP for the simultaneous detection of Cd^2+^ and Pb^2+^.

**Figure 2:**
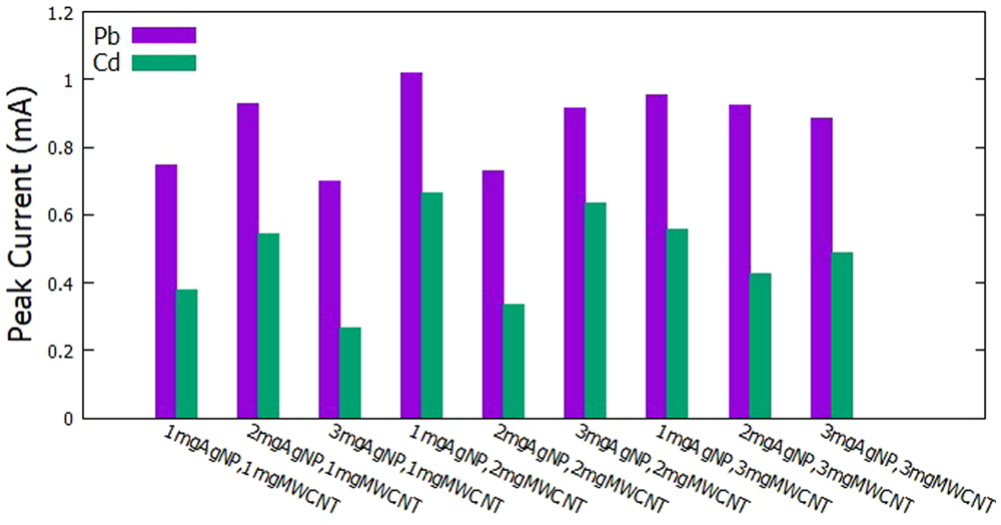
Anodic current peaks for varying AgNPs and MWCNTs for the simultaneous detection of Cd^2+^ and Pb^2+^.

The determination of the best modified GCE was done by varying the concentration of AgNPs and MWCNTs. The concentration of the MWCNTs was set at 1, 2, and 3 mg per 5 ml of Nafion. The concentration of the AgNPs was varied at 1, 2, and 3 mg per amount of MWCNT. The fabricated electrodes were then utilized to simultaneously detect constant amounts of Cd^2+^ and Pb^2+^. The following parameters were held constant: −0.9 V for the initial potential, 60 s for the deposition time, 30 s for the accumulation time, and 100 mV/s scan rate. The voltammograms obtained for the simultaneous detection of Cd^2+^ and Pb^2+^ are shown in Fig. 1 and the comparison of anodic current peaks for varying amounts of AgNPs and MWCNTs are shown in Fig. 2.

From the data gathered, it was observed that the electrode modified with 1 mg AgNP and 2 mg MWCNT exhibited the highest anodic peak currents for both Cd^2+^ and Pb^2+^, therefore, considered to be the amounts that will yield the optimum measurements for the heavy metals in the study.

### Optimization of ASV Parameters

In order to obtain the highest peak current of the determined best electrode for trace heavy metal detection, the parameters of anodic stripping voltammetry were optimized; that is, varying values of the deposition time, accumulation time and scan rate were used in simultaneous analyses of Cd^2+^ and Pb^2+^.

#### Deposition Time

**Figure 3:**
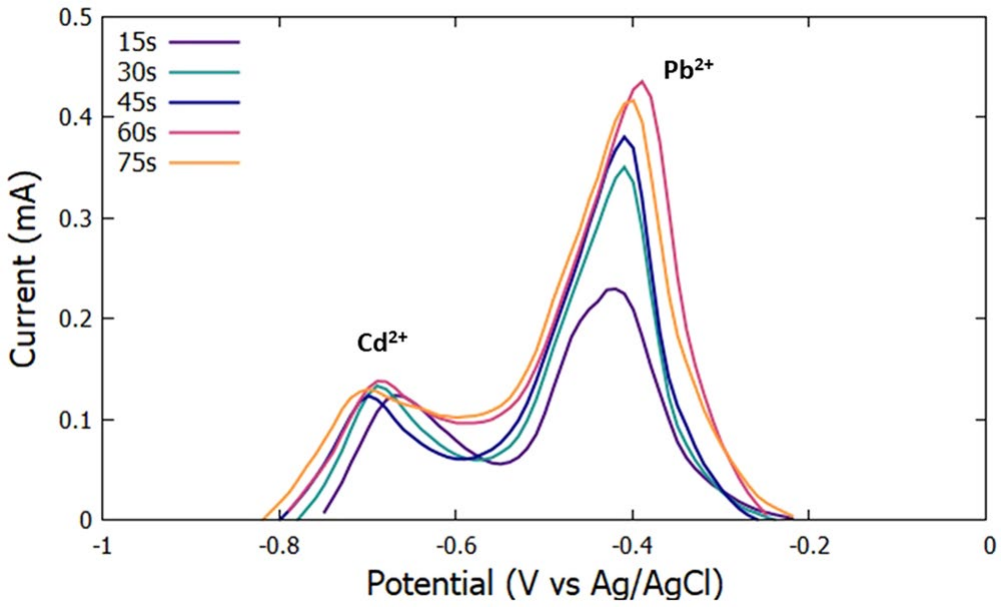
Voltammograms for the optimization of deposition time.

**Figure 4:**
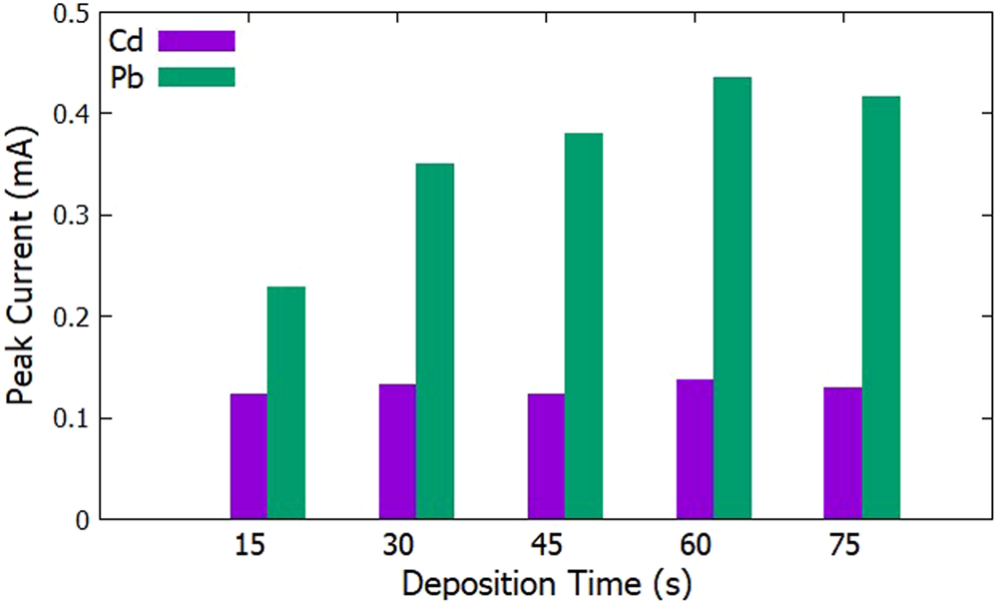
Anodic peak currents for varying deposition time for the simultaneous detection of Cd^2+^ and Pb^2+^.

The initial potential was set at −0.9 V, the accumulation period was set to 30 s, and the scan rate was set to 100 mV/s. The deposition time was varied at 15 s, 30 s, 45 s, 60 s, and 75 s. The highest anodic peak current for Cd^2+^ and Pb^2+^ was seen at a deposition time of 60 s (Figs 3 and 4).

#### Accumulation Time

**Figure 5:**
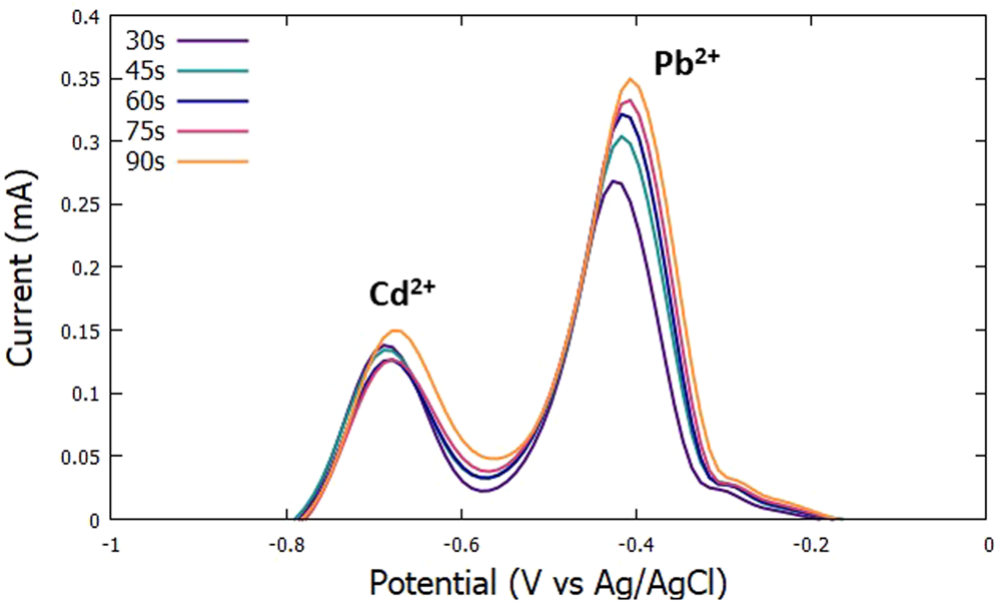
Voltammograms for the optimization of accumulation time.

**Figure 6:**
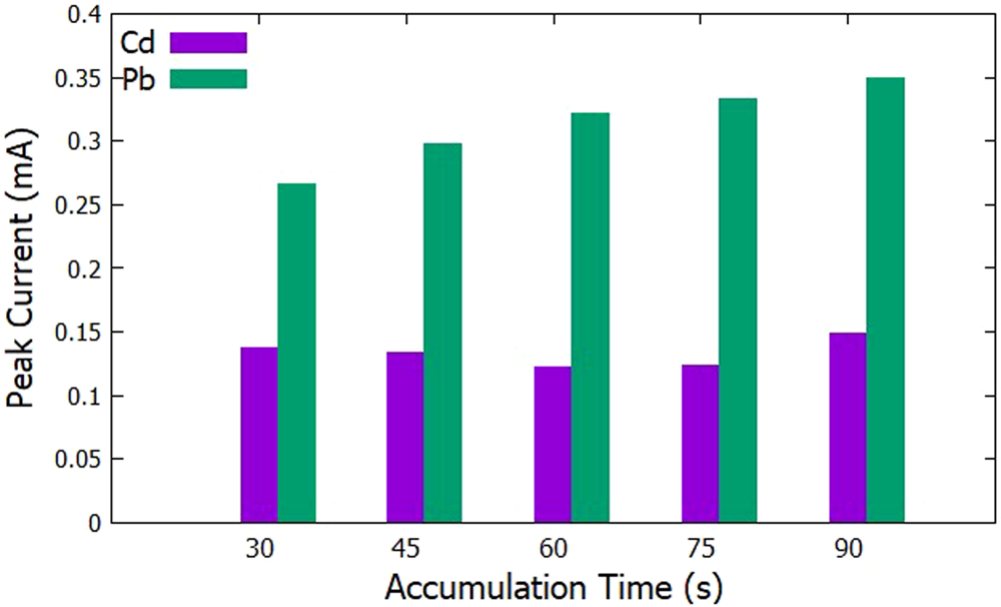
Anodic peak currents of Cd^2+^ and Pb^2+^ for varying accumulation time.

The initial potential was set at −0.9 V, the deposition time was set to 60 s since it was identified to be the optimized deposition time as seen previously in section 3.2.1, and the scan rate was set to 100 mV/s. The time for the accumulation period was varied at 30 s, 45 s, 60 s, 75 s, and 90 s. In Figs 5 and 6, it can be seen that the anodic current peaks for Pb^2+^ are increasing until the 90 s mark. On the other hand, the current peaks for Cd^2+^, was seen to decrease until the 60 s mark then increase again going to the 90 s mark. It can be observed that the highest current peak for both Cd^2+^ and Pb^2+^ can be seen at the 90 s mark. Therefore, 90 s was chosen to be the optimized accumulation time.

#### Scan Rate

**Figure 7:**
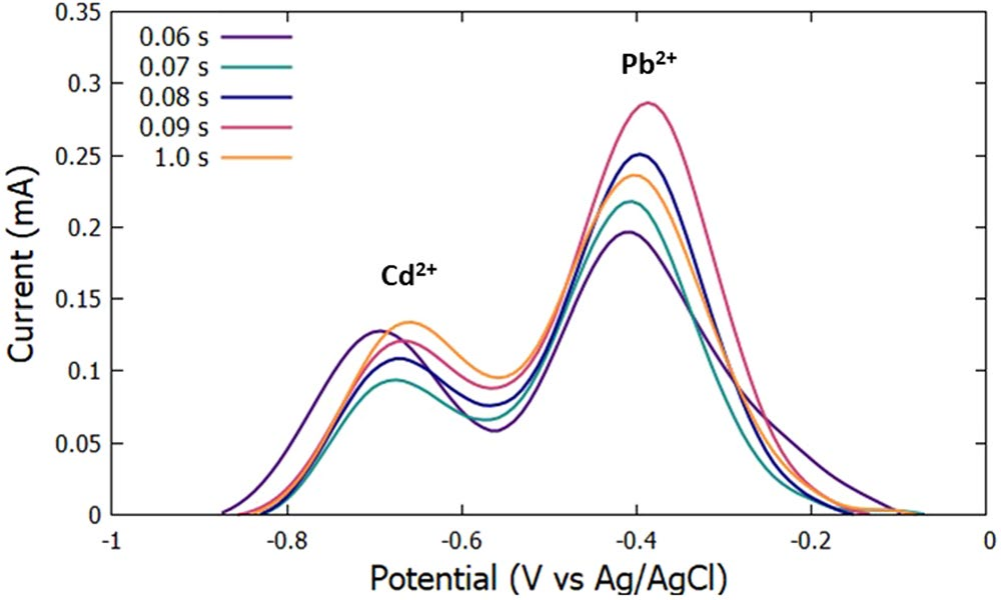
Voltammograms for the optimization of scan rate.

**Figure 8:**
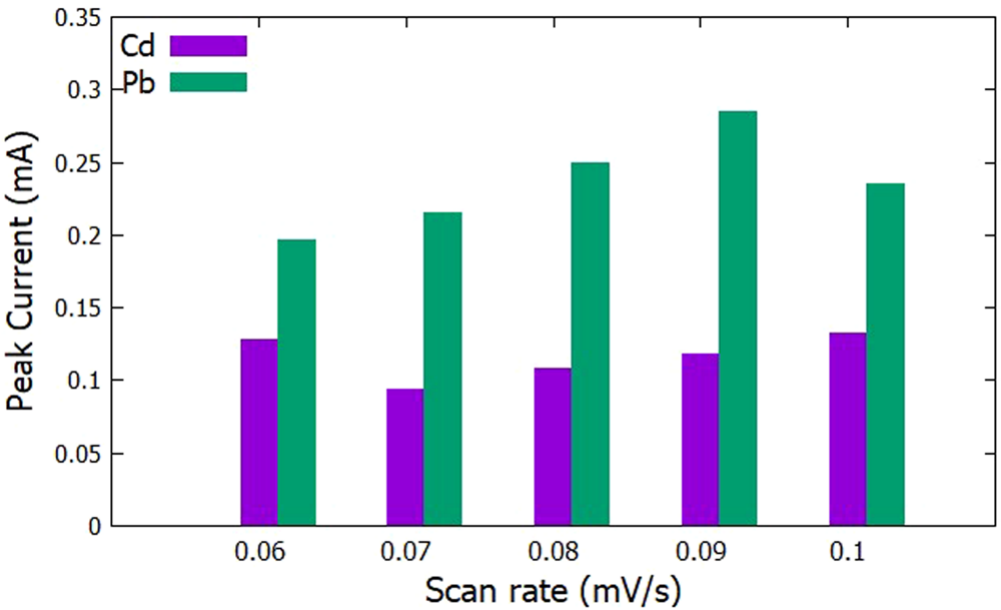
Anodic peak currents of Cd^2+^ and Pb^2+^for varying scan rate.

The initial potential was set at −0.9 V, 60 s deposition time, and 90 s accumulation time. The scan rate was then varied at 60 mV/s, 70 mV/s, 80 mV/s, 90 mV/s, and 100 mV/s. The highest anodic peak current for Cd^2+^ was obtained with a scan rate of 100 mV/s as seen in Figs 7 and 8. Since the fabricated electrode is less sensitive to Cd^2+^, the scan rate of 100 mV/s was chosen to be the optimum scan rate for the recipe since this gives the highest anodic peak current for Cd^2+^ and a fairly high anodic peak current for Pb^2+^ was obtained in that scan rate.

### Characterization

#### Electrochemical Characterization

**Figure 9:**
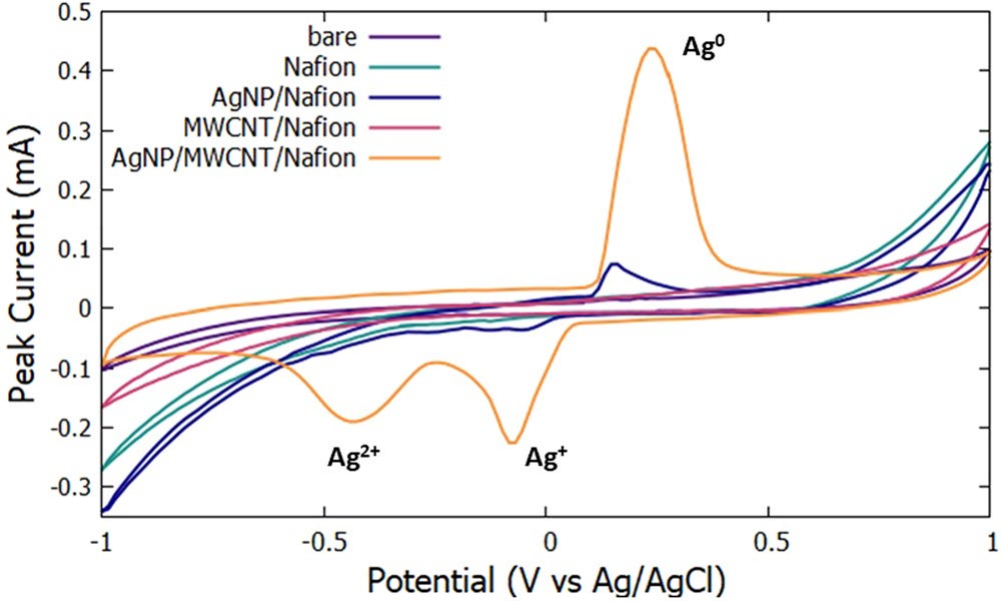
Cyclic voltammograms in 0.1 M NaCl at bare and modified electrodes at a scan rate of 0.09 V/s.

Cyclic voltammetry was employed to characterize the surface features of the bare and modified electrodes. Cyclic voltammograms for bare GCE, Nafion modified GCE, MWCNTs modified GCE, AgNPs modified GCE and AgNPs/MWCNTs/Nafion modified GCE in the potential range of −1.0 V to 1.0 V probed in a 0.1 M NaCl solution are shown in Fig. 9. Bare GCE, Nafion modified GCE and MWCNTs modified GCE do not show any anodic nor cathodic current peak in their respective voltammograms due to the absence of redox reaction on the surface of the electrode. Glassy carbon electrodes with AgNPs, however, exhibit anodic peaks at the 0.2 V potential in the forward scan and −0.1 V and −0.4 V in the reverse scan as the Ag oxidize and reduce, respectively. The oxidation peak refers to the oxidation of the Ag^+^ into Ag°, while the redox peak refers to the reduction of Ag° to Ag^+^. The mechanism for direct electron transfer (DET) is almost entirely provided by AgNPs^21^. Silver nanoparticle-modified GCE and MWCNT- modified GCE showed higher electrochemical response compared to the bare GCE and Nafion modified GCE due to the high electrical conductivity of AgNPs and MWCNTs. The AgNPs immobilized on the MWCNTs enhance the direct electron transfer due to the very high aspect ratio of the MWCNT. The highest electrochemical response observed was the AgNPs/MWCNTs/Nafion modified electrode verifying a homogenous dispersion of AgNPs and MWCNTs on the electrode surface.

On the forward scan of the cyclic voltammetry, a peak corresponding to Ag to Ag^+^ oxidation was observed with a peak potential of 0.25 V and a peak current of 0.45 mA, while on the reverse scan a reduction peak appeared at −0.1 V and −0.4 V with a peak current of −0.20 and −0.25 mA as shown in Fig. 9. The peaks in the reverse scan suggest that the Ag ° from the forward scan re-reduce to Ag^+^ and Ag^2+^ thus separating the peaks in its respective voltage potential^17^.

**Figure 10:**
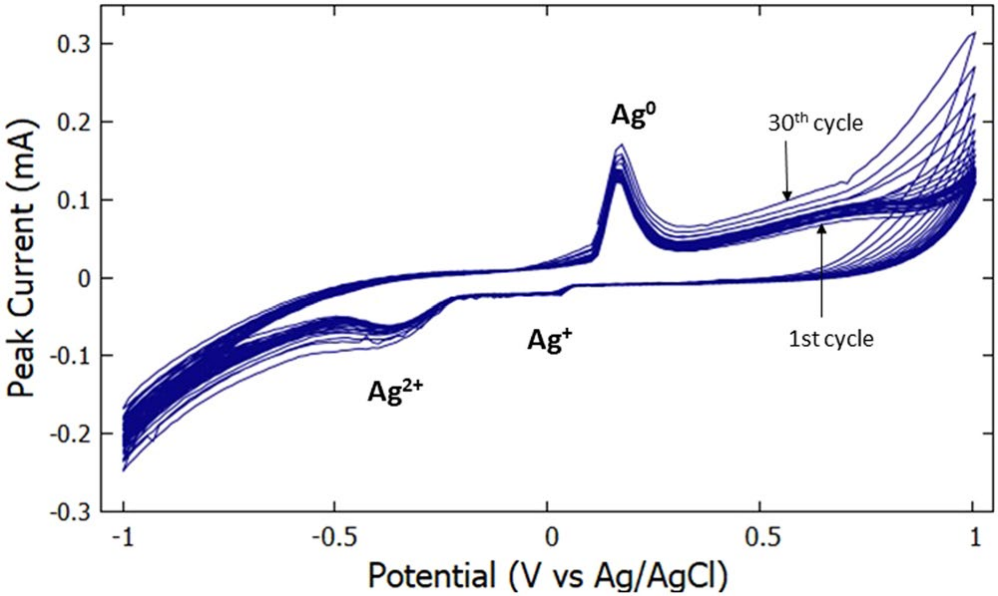
Successive voltammetric scans of GCE modified with AgNPs/MWCNTs/Nafion in 0.1 M NaCl solution at a scan rate of 0.09 V/s.

Cyclic voltammetry of 30 successive scans were carried on the AgNPs/MWCNTs/Nafion modified GCE in the potential range of −1.0 V to 1.0 V probed in a 0.1 M NaCl solution shown in Fig. 10. The integrity of the modified electrode was observed to be excellent as the successive scans exhibited diminutive deterioration from the first scan up to the thirtieth scan. This suggests that the fabricated electrode has a substantial surface area and its active surface is not completely covered^22^.

#### Morphology

**Figure 11:**
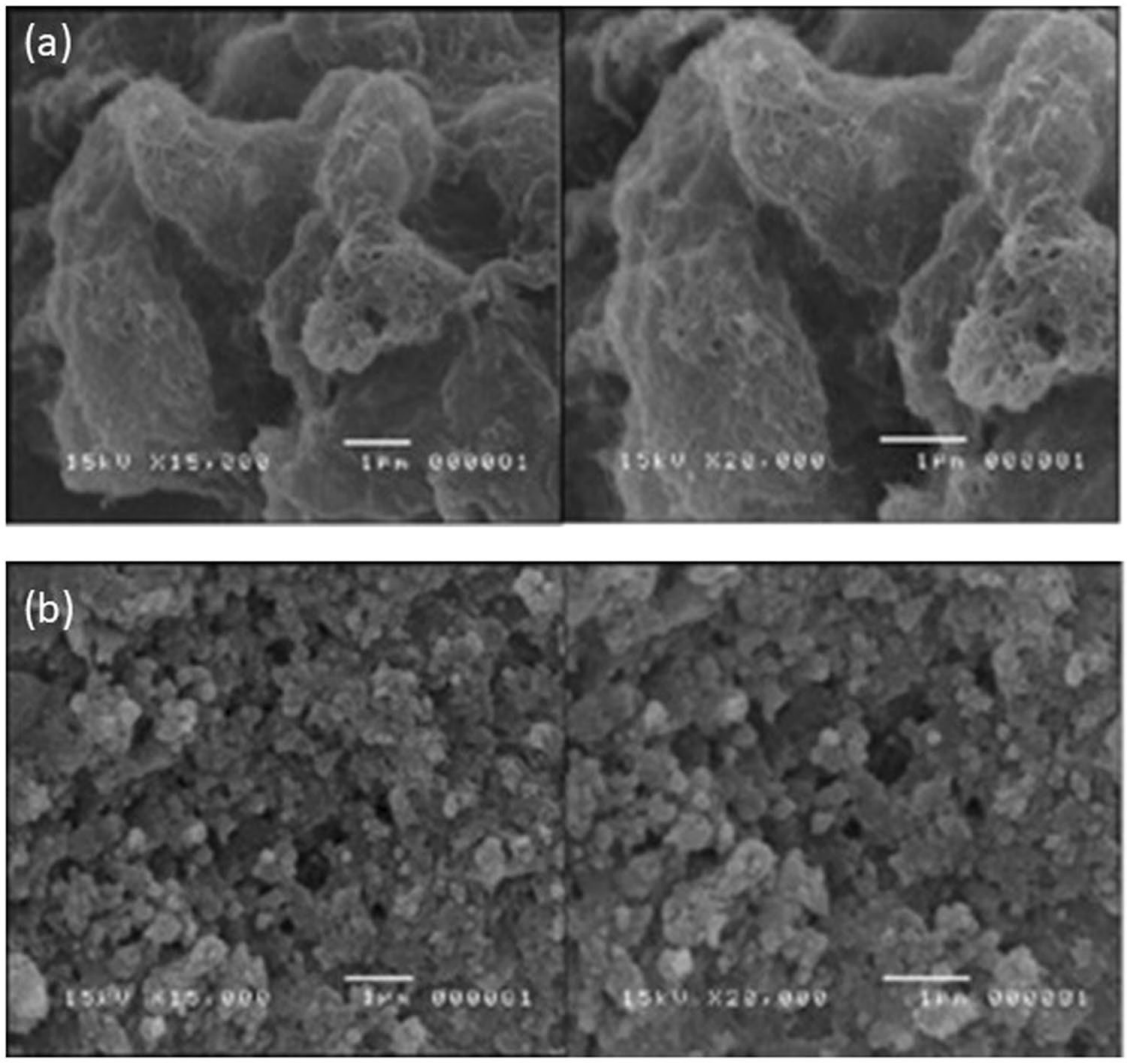
SEM images of (**a**) MWCNTs/Nafion modified GCE and (**b**) AgNPs/Nafion modified GCE.

The AgNPs/Nafion and MWCNTs/Nafion were characterized individually using scanning electron microscopy (SEM). The SEM micrographs showed that the MWCNTs were indeed multi-walled as they are composed of multiple rolled layers of graphene (*cf*. Figure 11a). The morphology of the AgNPs appear to be truncated octahedrons as seen in Fig. 11b in agreement with simulated AgNPs of Zhou *et al*.^23^. The AgNP and MWCNT both have high surface area, high electrical and thermal conductivity and high chemical stability, which allowed the electrode to increase its electrochemical capacity^24^. Each modifier was seen to be dispersed uniformly on the surface of each GCE.

**Figure 12:**
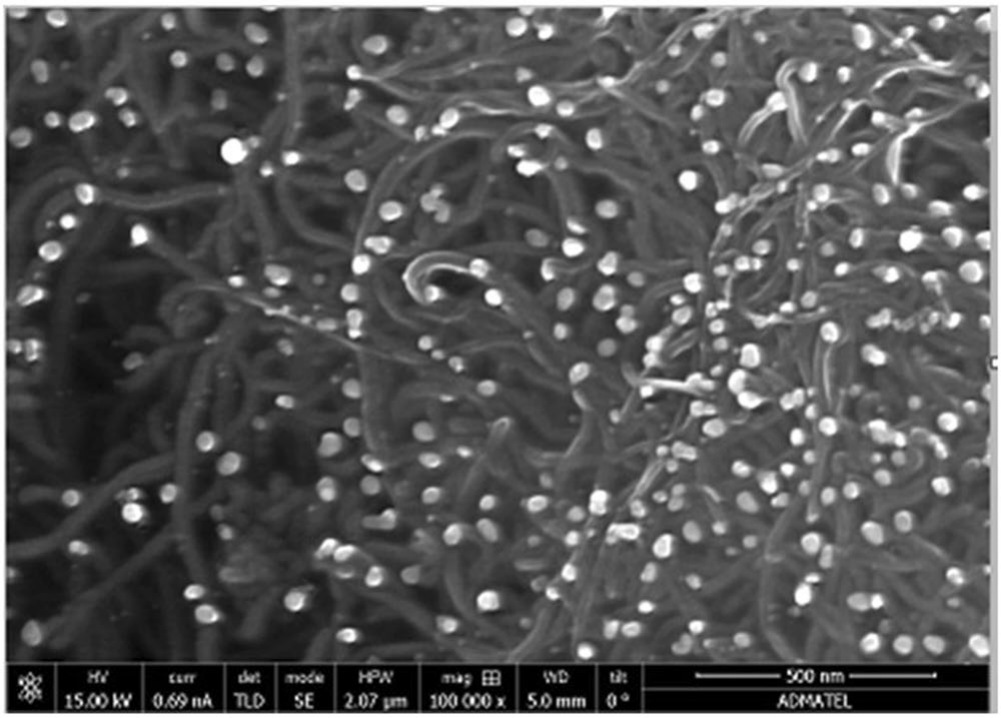
FESEM image of AgNP/MWCNT/Nafion modified GCE.

**Figure 13:**
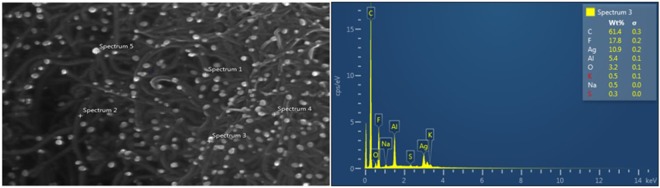
FESEM with EDX point analysis of modified electrode surface.

The morphological characterization of the modified electrode was performed with field emission scanning electron microscopy (FESEM-EDX). The FESEM-EDX analysis exhibited the morphological characteristics of both AgNPs and MWCNTs respectively. The FESEM image shown in Fig. 12 revealed that the drop coated AgNPs/MWCNTs/Nafion were dispersed homogeneously on the electrode surface. The prolonged ultrasonication of the modifier solution allowed the AgNPs, MWCNTs and Nafion to amalgamate, thus embedding the AgNPs to the MWCNTs. The resulting electrode modifier solution has both the characteristics of the AgNPs and MWCNTs which resulted in the increase of the electrochemical capabilities of the modified electrode. In the EDX point analysis of the AgNPs/MWCNTs/Nafion modified electrode, the purity of the AgNPs, MWNTs and Nafion were verified as shown in Fig. 13. High carbon percentage is attributed to the MWCNTs component of the modified electrode. The MWCNTs size, shape and weight are substantial factors in the high carbon percentage in the analysis. In spectrum 3, 10.9% of Ag was observed which proved that the circular particles that are lodged on the walls of the MWCNTs are the AgNPs. The Nafion component of the modified electrode is responsible for the remaining elements such as fluorine (F), oxygen (O) and sulfur (S). The routine cleaning of the electrode can be attributed to the presence of aluminum (Al) in the EDX point analysis as alumina slurry was utilized in the process. The presence of sodium (Na) and potassium (K) are due to the inherent impurities of the GCE^25^.

### Calibration Curve

**Figure 14:**
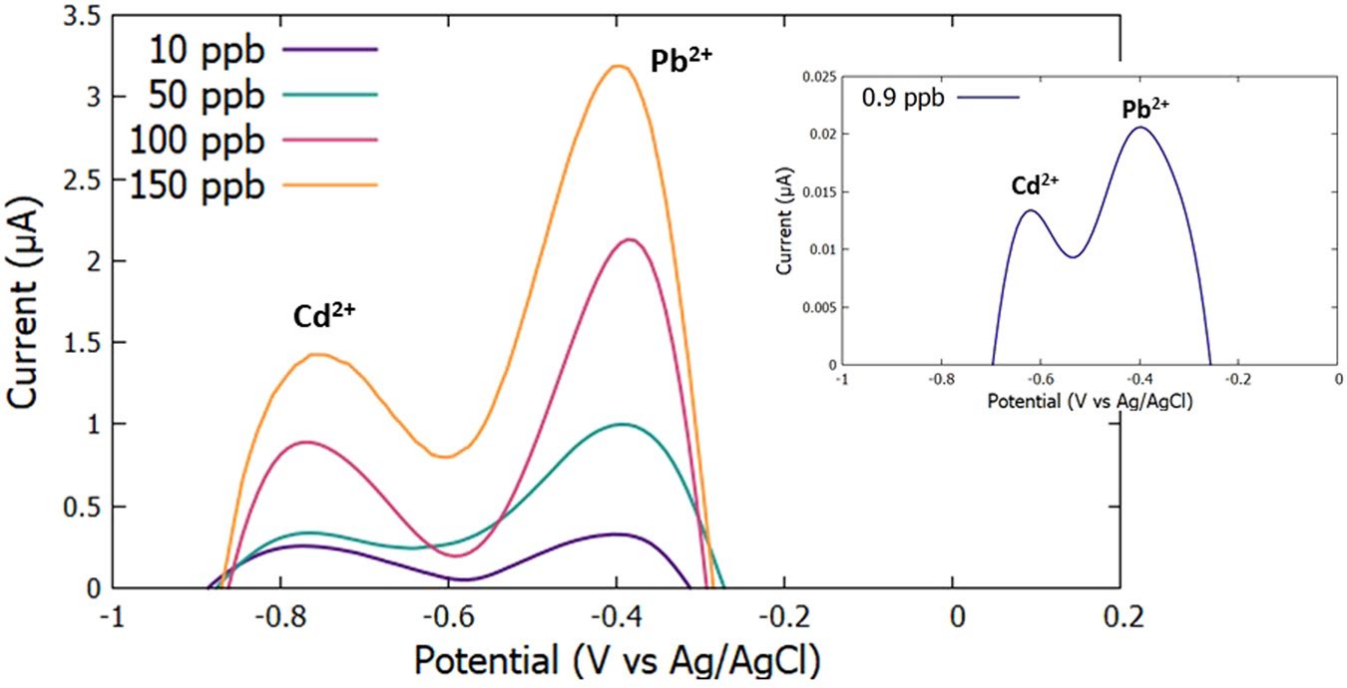
Voltammograms of varying Pb^2+^ and Cd^2+^ concentrations from 0.9 ppb to 200 ppb.

The calibration curves were obtained by varying the analyte concentrations from 0.9 ppb to 150 ppb for both PbCl_2_ and CdCl_2_ (Fig. 14). The heavy metal concentrations were then plotted against the corresponding current peaks to obtain the calibration curve for each metal.

**Figure 15:**
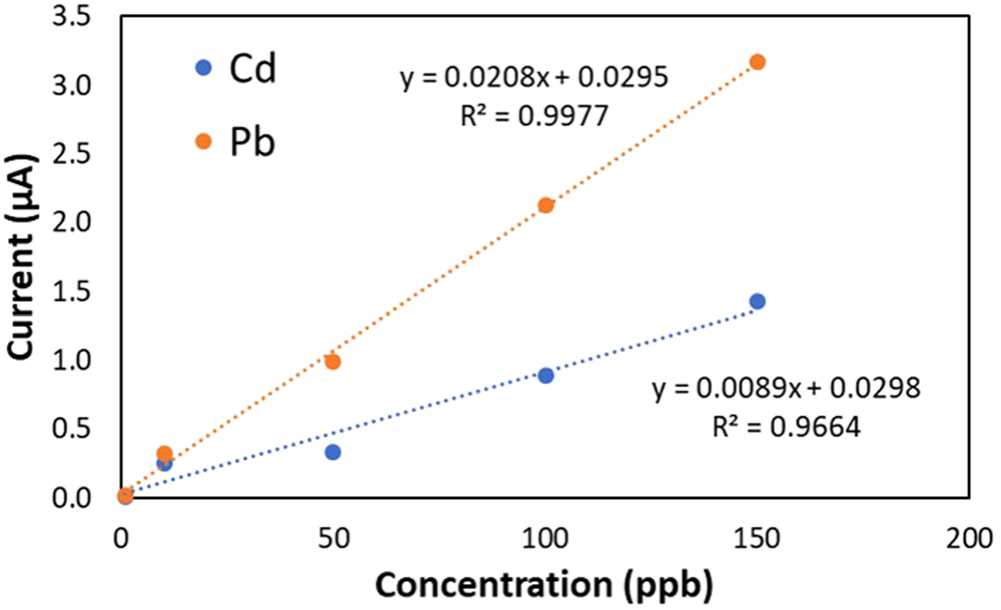
Calibration curves for Pb^2+^ and Cd^2+^.

Figure 15 shows the calibration curves of Pb^2+^ and Cd^2+^, respectively. The obtained Pearson’s coefficient indicates a strong linear relationship between the reduction current and the heavy metal concentration.

### Real Sample Analysis

#### Anodic Stripping Voltammetry

Using the best modified electrode, 1 mg AgNP and 2 mg MWCNT, and the optimized parameters of initial potential of −0.9 V, deposition time of 75 s, accumulation time of 90 s, and scan rate of 100 mV/s, that was determined from the voltammograms that yielded the highest anodic peak currents for Pb^2+^ and Cd^2+^, real sampling analysis was done using ASV.

**Figure 16:**
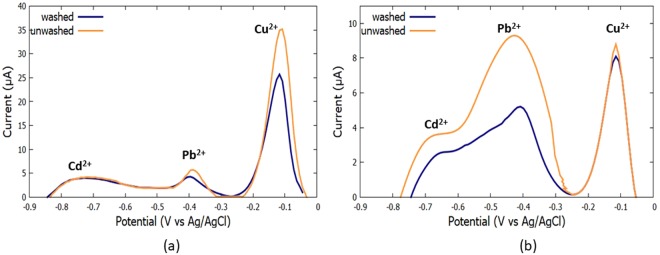
Voltammograms of (**a**) organic camote tops and (**b**) organic squash.

Table 1 shows the different heavy metal concentrations in both organic (washed and unwashed) and non-organic (washed and unwashed) vegetables. It was seen that in both organic and non-organic vegetables, kabocha or the common Philippine squash (*Cucurbita maxima*) contains the highest concentration of lead with 376.85 ppb and 449.08 ppb, respectively. On the other hand, the vegetable with the least lead content was the organic Bok Choy (*Brassica rapa subsp*. *chinensis*) with 96.80 ppb and the non-organic cabbage (*Brassica rapa subsp*. *chinensis)* with 264.60 ppb. The vegetables with the highest cadmium content in both organic and non-organic samples are the camote tops (*Ipomoea batatas*) with 452.01 ppb and 696.78 ppb, respectively. The vegetable with the least cadmium content in both organic and non-organic sample is Bok Choy (*Brassica rapa subsp*. *chinensis*) with 59.04 ppb and 125.22 ppb, respectively. It was observed that organic vegetables contain less concentration of both Pb^2+^ and Cd^2+^ compared to their non-organic counterparts. This is to be expected since organic vegetables are less exposed to environmental pollution and are devoid of agricultural pesticides. However, due to the illegal gold mining present in Sitio San Ysiro, the organic vegetables were seen to contain unknown copper concentrations, as shown in Fig. 16. Copper is a known mine tailing of gold mining^26^. There were reports of multiple small scale illegal gold mining conducted by an anonymous group within the city of Antipolo that includes Purok Libis, Purok Casunugan and Sitio San Ysiro^13,27^. It can also be seen that the heavy metal content in unwashed vegetables is indeed higher compared to the heavy metal content of the washed vegetable, which agrees with Locatelli and Melucci^11^. The high heavy metal content may be ascribed to environmental pollution caused by anthropogenic activities, and the frequent spraying of pesticides prior to harvest of the vegetables (for non-organic samples)^11^. It can be seen that all the vegetables in this study contain Pb^2+^ and Cd^2+^ concentration way above the WHO toxicity limits. Therefore, all the vegetables used in this study, both organic and non-organic, may be deemed to be toxic for human consumption.

**Table 1:** **C**oncentrations (ppb) of Pb^2+^ and Cd^2+^ in Vegetables using ASV.

Heavy Metals	Camote Tops (Ipomoea batatas)				Kabocha Squash (Cucurbita Maxima)			
	Organic Unwashed	Organic Washed	Non-organic Unwashed	Non-organic Washed	Organic Unwashed	Organic Washed	Non-organic Unwashed	Non-organic Washed
Cd	452.01	442.22	696.78	496.11	440.33	280.11	548.95	327.71
Pb	284.39	212.00	414.05	397.05	376.85	258.95	492.08	465.09
**Heavy Metals**	**Cabbage (Braccica oleracea var. capitala)**				**Bokchoy (Brassica rapa subsp. Chinensis)**			
	**Organic Unwashed**	**Organic Washed**	**Non-organic Unwashed**	**Non-organic Washed**	**Organic Unwashed**	**Organic Washed**	**Non-organic Unwashed**	**Non-organic Washed**
Cd	380.78	198.89	538.78	302.78	93.71	59.04	233.89	125.22
Pb	161.05	122.50	264.60	155.35	96.80	38.89	312.50	177.90
**Heavy Metals**	**Soil**				**Water**			
Cd	253.43				484.22			
Pb	338.44				154.10

#### Atomic Absorption Spectroscopy (AAS)

To verify the ASV results of the vegetable samples, AAS was utilized to detect cadmium and lead. A standard of concentration ranging from 200 ppb to 1000 ppb for lead and cadmium was used to obtain a calibration curve for the AAS analysis. Table 2 displays the different concentrations of Pb^2+^ and Cd^2+^ of both organic and non-organic vegetables. The same trend that can be seen in Table 1 can be observed in Table 2, that organic vegetables contain less heavy metal concentrations compared to the non-organic ones and that the heavy metal concentrations in the sample decrease when they are washed.

**Table 2:** Concentrations (ppb) of Pb^2+^ and Cd^2+^ using AAS.

Heavy Metals	Camote Tops (Ipomoea batatas)				Kabocha Squash (Cucurbita Maxima)			
	**Organic Unwashed**	**Organic Washed**	**Non-organic Unwashed**	**Non-organic Washed**	**Organic Unwashed**	**Organic Washed**	**Non-organic Unwashed**	**Non-organic Washed**
Cd	145.00	177.50	130.00	496.11	76.75	66.750	106.75	86.75
Pb	360.00	260.00	430.00	370.00	200.00	180.00	310.00	270.00
**Heavy Metals**	**Cabbage (Braccica oleracea var. capitala)**				**Bokchoy (Brassica rapa subsp. Chinensis)**			
	**Organic Unwashed**	**Organic Washed**	**Non-organic Unwashed**	**Non-organic Washed**	**Organic Unwashed**	**Organic Washed**	**Non-organic Unwashed**	**Non-organic Washed**
Cd	145.00	82.50	177.50	125.00	127.50	90.00	152.50	135.00
Pb	270.00	210.00	430.00	290.00	320.00	290.00	480.00	390.00
**Heavy Metals**	**Soil**				**Water**			
Cd	502.50				555.00			
Pb	1440.00				130.00			

#### Comparison of Results

Lead and cadmium concentrations resulting from ASV and AAS analysis were compared through percentage errors and percentage difference. The results show that there is a noticeable disparity in the concentrations of both metal ions detected by AAS as well as ASV as seen in Table 3. The disparity can be ascribed to the filtered acid digested samples having suspended particles that may have partially blocked the nebulizer of the atomic absorption spectrophotometer. With the nebulizer partially blocked, lower absorbance resulted yielding a lower concentration of Pb^2+^ and Cd^2+^^27^. The concentrations of Pb^2+^ and Cd^2+^ observed in ASV are higher than the observed values in AAS. The AAS spectra in this study were obtained using an AA-6300 Shimadzu Atomic Absorption Spectrophotometer. This model can only detect a minimum ~85 ppb^28^. The lowest concentration detected via AAS in this study was 82.5 ppb. The modified electrode fabricated in this study was able to detect down to 1.80 ppb via ASV. This makes the AgNP/MWCNT/Nafion modified GCE fabricated in this study a superior sensor for detecting trace heavy metals such as Pb^2+^ and Cd^2+^. It allows highly sensitive detection down to 216 parts per trillion for Pb^2+^ and 481 parts per trillion for Cd^2+^. In terms of selectivity, ASV is more versatile as it can detect a variety of heavy metals in one sweep unlike AAS in that it can detect one element at a time and that one has to know what is to be detected beforehand.

**Table 3:** Comparison of lead and cadmium concentrations using ASV and AAS.

Real Samples	Pb			Cd		
	ASV Concentration (ppb)	AAS Concentration (ppb)	% Diff	ASV Concentration (ppb)	AAS Concentration (ppb)	% Diff
TOW	212.00	260.00	20.34	442.22	117.50	116.03
TOU	284.39	360.00	23.47	452.01	145.00	102.85
TNW	397.05	370.00	7.05	496.11	130.00	116.95
TNU	411.05	430.00	4.51	696.78	177.50	118.79
COW	122.50	210.00	52.63	198.89	82.50	82.72
COU	161.05	270.00	50.55	440.33	145.00	89.69
CNW	155.35	290.00	60.47	302.78	125.00	83.12
CNU	264.60	430.00	2.02	538.78	177.50	121.05
SOW	258.95	180.00	35.97	280.11	66.75	123.02
SOU	376.85	200.00	61.32	440.33	76.75	140.63
SNW	465.09	270.00	53.08	327.71	86.75	116.28
SNU	492.08	310.00	45.40	548.95	106.75	134.88
BOW	38.89	290.00	152.70	59.04	90.00	41.54
BOU	96.80	320.00	107.10	93.71	127.50	30.55
BNW	177.90	390.00	74.70	125.22	135.00	7.51
BNU	312.50	480.00	42.27	233.89	152.50	42.13
Soil	253.43	1440.0	123.88	484.22	502.50	123.88
Water	338.44	130.00	65.90	154.10	555.00	65.90

Legend: TOW- organic washed camote tops; TOU - organic unwashed camote tops; TNW- non-organic washed camote tops; TNU – organic unwashed camote tops; COW – organic washed cabbage; COU– organic unwashed cabbage; CNW – non-organic washed cabbage; CNU – non-organic unwashed cabbage; SOW- organic washed squash; SOU - organic unwashed squash; SNW – non-organic washed squash; SNU – non-organic unwashed squash; BOW– organic washed Bok choy; BOU – organic unwashed Bok choy; BNW – non-organic washed Bok choy; BNU – non-organic unwashed Bok choy.

### Limit of Detection and Limit of Quantification

As seen in Table 4, the lowest limit of detection of the modified electrode used in this study is 216 parts per trillion for Pb^2+^ and 481 parts per trillion for Cd^2+^. When compared to the AAS, it can be seen that the fabricated electrode is more sensitive than the AAS since the limit of detection of AAS was 1.17 parts per million (ppm) for Pb^2+^ and 0.94 ppm for Cd^2+^ based on the AAS calibration curves. The data obtained agrees with Locatelli and Melucci and Abadin, *et al*.^11,29^. The fabricated electrode used in this study is more than capable of detecting Pb^2+^ and Cd^2+^ concentrations below the WHO limit of toxicity.

**Table 4:** ASV and AAS Limit of Detection and Limit of Quantification.

	SD_ASV_	SD_AAS_	m_ASV_	m_AAS_	ASV Experimental LOD (ppb)	ASV Theoretical LOD (ppb)	AAS Theoretical LOD (ppb)	ASV LOQ (ppb)	AAS LOQ (ppb)
Pb	3.69 × 10^−2^	3.54 × 10^−3^	2.00 × 10^−8^	1.00 × 10^−5^	0.49	0.22	1169.76	0.66	3544.71
Cd	3.69 × 10^−2^	1.15 × 10^−2^	9.00 × 10^−9^	4.00 × 10^−5^	1.86	0.48	944.66	1.87	2862.61

### Analytical Sensitivity

The analytical values for the varied concentrations of Cd^2+^ and Pb^2+^ are seen in Table 5. It analytical sensitivity increases as the concentration of the heavy metal increases.

**Table 5:** Analytical Sensitivity.

	Concentration (ppb)	Current (A)	SD (A)	Slope (A/ppb)	Analytical Sensitivity (1/ppb)
Cd	1.864	1.32 × 10^−8^	6.38 × 10^−7^	9.00 × 10^−9^	1.41 × 10^−2^
	25.16	2.56 × 10^−7^	6.27 × 10^−7^	9.00 × 10^−9^	1.44 × 10^−2^
	33.92	3.35 × 10^−7^	6.22 × 10^−7^	9.00 × 10^−9^	1.45 × 10^−2^
	95.52	8.90 × 10^−7^	5.44 × 10^−7^	9.00 × 10^−9^	1.65 × 10^−2^
	155.1	1.43 × 10^−6^	3.70 × 10^−7^	9.00 × 10^−9^	2.43 × 10^−2^
Pb	0.494	2.01 × 10^−8^	1.50 × 10^−6^	2.00 × 10^−8^	1.34 × 10^−2^
	14.78	3.26 × 10^−7^	1.42 × 10^−6^	2.00 × 10^−8^	1.41 × 10^−2^
	47.88	9.88 × 10^−7^	1.37 × 10^−6^	2.00 × 10^−8^	1.46 × 10^−2^
	104.6	2.12 × 10^−8^	1.26 × 10^−6^	2.00 × 10^−8^	1.59 × 10^−2^
	157.2	3.17 × 10^−6^	9.31 × 10^−7^	2.00 × 10^−8^	2.15 × 10^−2^

### Calibration Sensitivity

Table 6 shows the calibration sensitivity of the two methods used in this study. It can be observed that the calibration sensitivity is greater in AAS than ASV. This suggests that AAS is more sensitive than ASV^17^. This only shows that the response is more prominent for every increment in concentration with AAS than with ASV. This is the only advantage of AAS over ASV.

**Table 6:** Calibration Sensitivity of AAS and ASV.

	Calibration Sensitivity	
	m_ASV_ (A/ppb)	m_AAS_ (A/ppb)
Pb	2 × 10^−8^	1 × 10^−5^
Cd	9 × 10^−9^	4 × 10^−5^

### Comparison with Other Works

Table 7 shows the comparison of the fabricated electrode in this study with previous works. It can be concluded that the electrode in this study is far superior than most of the electrodes fabricated in previous works.

**Table 7:** Performance Comparison with Other Works.

Electrode	Method	Real Samples	Heavy Metals Detected	LOD (ppb)	Reference
Mercury film	Inverse voltammetry	Vegetables	Cu, Cd, Pb, Zn	Cu = 5000 Cd = 30 Pb = 50 Zn = 1000	^5^
Nafion- coated Bi film	DPASV	Vegetables	Cd, Pb, Zn	Cd = 17 Pb = 0.17 Zn = 0.30	^6^
Hanging mercury drop	SWASV	Vegetables	Hg, Cu, Pb, Cd, Zn	Hg = 0.081 Cu = 0.18 Pb = 0.12 Cd = 0.19 Zn = 0.23	^11^
AgNP modified GCE	Linear Sweep Voltammetry	Water	Hg	Hg = 0.028	^17^
MWCNT/Nafion nanocomposite film	SWASV	Water	Cd	Cd = 1	^30^
Bi/MWCNT/EBP/Nafion modified GCE	SWASV	Soil	Cd, Pb	Cd = 0.08 Pb = 0.06	^31^
Engineered MWCNT	SWASV	Water	Cd, Pb	Cd = 0.4 Pb = 0.3	^32^
AuNP/Graphene./Nafion modified GCE	ASV	Cigarettes	Cd, Pb, Cu	Cd = 0.122 Pb = 0.014 Cu = 209	^33^
AuNP/[Ru(NH_3_)_6_]^3+^./Nafion modified GCE	ASV	Hair Dyes	Cd, Pb	Cd = 200 Pb = 45	^34^
AgNPs/MWCNTs/Nafion modified GCE	ASV	Vegetables	Cd, Pb	Cd = 0.481 Pb = 0.216	This work

## Conclusions

The fabricated AgNPs/MWCNTs/Nafion modified glassy carbon electrodes were successful in determining trace concentraions of Cd^2+^ and Pb^2+^. Morphological characterization of the modified GCEs by SEM and EDX showed that the modifier solution dispersed homogeneously on the electrode surface henceforth increasing the electrochemical capabilities of the modified electrode due to the characteristics brought about by MWCNTs and AgNPs. Electrochemical characterization using cyclic voltammetry suggests that the modified CGE have high electrochemical response in a NaCl solution as the anodic peak current reaches up to 0.45 mA. Out of the nine modifier contents used, 1 mg AgNP and 2 mg MWCNT exhibited the highest anodic peak currents for lead and cadmium. All calibration curves obtained using the best modified electrode showed a linear relationship between heavy metal concentration and peak current and the detection limits were found to be 0.216 ppb for Pb^2+^ and 0.481 ppb for Cd^2+^. Real sampling analysis was carried out using organic and non-organic vegetables. Trace amounts of lead, cadmium, and copper were detected in both organic and non-organic vegetable samples. The heavy metal concentrations of washed vegetables were less compared to those of unwashed vegetables.
